# Effects of Triazole Derivatives on Strigolactone Levels and Growth Retardation in Rice

**DOI:** 10.1371/journal.pone.0021723

**Published:** 2011-07-08

**Authors:** Shinsaku Ito, Mikihisa Umehara, Atsushi Hanada, Nobutaka Kitahata, Hiroki Hayase, Shinjiro Yamaguchi, Tadao Asami

**Affiliations:** 1 Department of Applied Biological Chemistry, The University of Tokyo, Tokyo, Japan; 2 RIKEN Plant Science Center, Yokohama, Japan; Seoul National University, Republic of Korea

## Abstract

We previously discovered a lead compound for strigolactone (SL) biosynthesis inhibitors, TIS13 (2,2-dimethyl-7-phenoxy-4-(1*H*-1,2,4-triazol-1-yl)heptan-3-ol). Here, we carried out a structure-activity relationship study of TIS13 to discover more potent and specific SL biosynthesis inhibitor because TIS13 has a severe side effect at high concentrations, including retardation of the growth of rice seedlings. TIS108, a new TIS13 derivative, was found to be a more specific SL biosynthesis inhibitor than TIS13. Treatment of rice seedlings with TIS108 reduced SL levels in both roots and root exudates in a concentration-dependent manner and did not reduce plant height. In addition, root exudates of TIS108-treated rice seedlings stimulated *Striga* germination less than those of control plants. These results suggest that TIS108 has a potential to be applied in the control of root parasitic weeds germination.

## Introduction

Strigolactones (SLs) are a group of terpenoid lactones that have been found in root exudates of various plant species and were first identified as seed germination stimulants of root parasitic weeds such as *Orobanche* and *Striga* [1]. SLs also induce hyphal branching in arbuscular mycorrhizal fungi that form symbiotic associations with the roots of more than 80% of land plants [2]. More recently, two groups reported that SLs or their metabolites inhibit shoot branching [3], [4]. Genetic analysis of a series of branching mutants, *more axillary growth* (*max*) mutants of *Arabidopsis*, *ramosus* (*rms*) mutants of pea, *dwarf* (*d*) or *high tillering dwarf* (*htd*) mutants of rice and *decreased apical dominance* (*dad*) mutants of petunia, revealed that the biosynthesis of SLs is mediated by two carotenoid cleavage dioxygenases, CCD7 (MAX3, RMS5 D17/HTD1, DAD3) and CCD8 (MAX4, RMS1, D10, DAD1), one cytochrome P450 monooxygenase (MAX1) and one novel iron-containing protein (D27) [5], [6]. However, several biosynthetic steps still need to be uncovered [7].

Root parasitic weeds are considered as harmful plants in sub-Saharan Africa, the Middle East and Asia that parasitize the roots of host plants. It was reported that around 300 million people are affected by *Striga* in Africa with a loss of $US 7 to 10 billion [8]. Although many approaches to control the parasitic weeds have been explored, useful solutions have not been discovered.

Biosynthetic inhibitors of biologically active substances can control their endogenous levels in developmental stages and sites of various plants. In addition, even if a mutation or targeted knockout of an individual gene in sets of paralogous genes could affect phenotypes to a small degree, biosynthetic inhibitors can overcome such gene redundancy in many cases. Therefore, the use of specific biosynthesis inhibitors is an alternative and valuable way to determine the physiological functions of endogenous substances. As was seen for brassinosteroid biosynthesis inhibitors [9]–[12], specific SL biosynthesis inhibitors would be useful tools both for functional studies of SL biosynthesis and for the assessment of the effect of SLs in plants. Moreover, SL biosynthesis inhibitors can be applicable for the regulation of germination and infestation of root parasitic weeds.

To discover new SL biosynthesis inhibitors, we screened a chemical library of triazole derivatives, because several triazole-containing chemicals have previously been shown to act as efficient inhibitors of cytochrome P450 monooxygenases and the proposed SL biosynthesis pathway involves at least one cytochrome P450 (CYP711A). We discovered one SL biosynthesis inhibitor, 2,2-dimethyl-7-phenoxy-4-(1*H*-1,2,4-triazol-1-yl)heptan-3-ol (TIS13) [13]. TIS13 treatment of rice seedlings reduces SL levels in both roots and root exudates and reduces the *Striga* germination-stimulating activity in root exudates. However, TIS13 treatment strongly reduces plant height that is not caused by the inhibition of SL biosynthesis, suggesting the inhibition of the biosynthesis of other phytohormones such as gibberellin and/or brassinosteroid. To develop specific SL biosynthesis inhibitors, it is necessary to weaken this side effect. In this context, we synthesized several TIS13 analogues and evaluated their ability to inhibit SL biosynthesis and their side effects.

## Results and Discussion

### Synthesis of TIS13 derivatives

**Figure 1:**
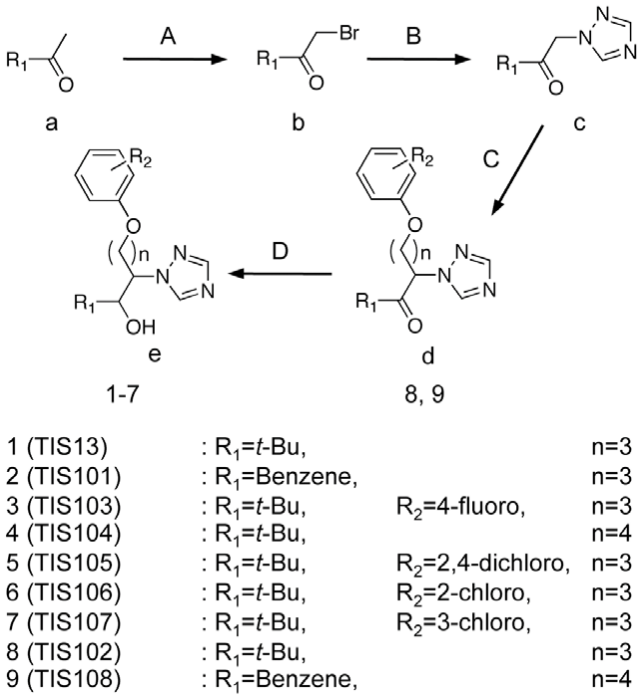
Syntheses of designed chemicals. (A) Br_2_, AlCl_3_, ether; (B) 1,2,4-triazole, K_2_CO_3_, acetone; (C) R_2_X, 60% NaH, DMF, reflux; (D) NaBH_4_, MeOH.

To find specific inhibitors for SL biosynthesis, we performed a structure-activity relationship study. Fig. 1 shows the structures of TIS13 and TIS13 derivatives that were tested in this study. All chemicals were synthesized according to a previous report [14] as follows. Bromine was introduced to the α-position of ketone (**a**) to give α-bromoketone (**b**), which is coupled with triazole under basic conditions to give **c**, which in turn gave **d** following aprotic and basic conditions. Subsequent reduction with sodium borohydride gave **e**.

### Effects of TIS13 derivatives on 2′-epi-5-deoxystrigol (epi-5DS) levels and growth retardation

**Figure 2:**
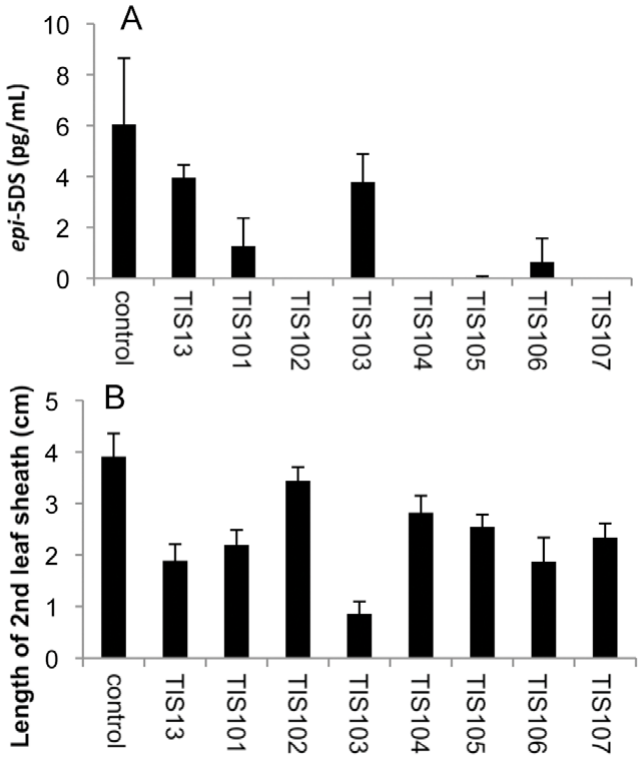
Effect of chemicals on 2-week-old rice seedlings. (A) *epi*-5DS levels in root exudates of 1 µM chemical-treated rice seedlings determined by LC-MS/MS. The data are means ± SD of three samples. (B) The length of 2nd leaf sheath in 10 µM chemical-treated rice. The data are means ± SD of six samples.

**Figure 3:**
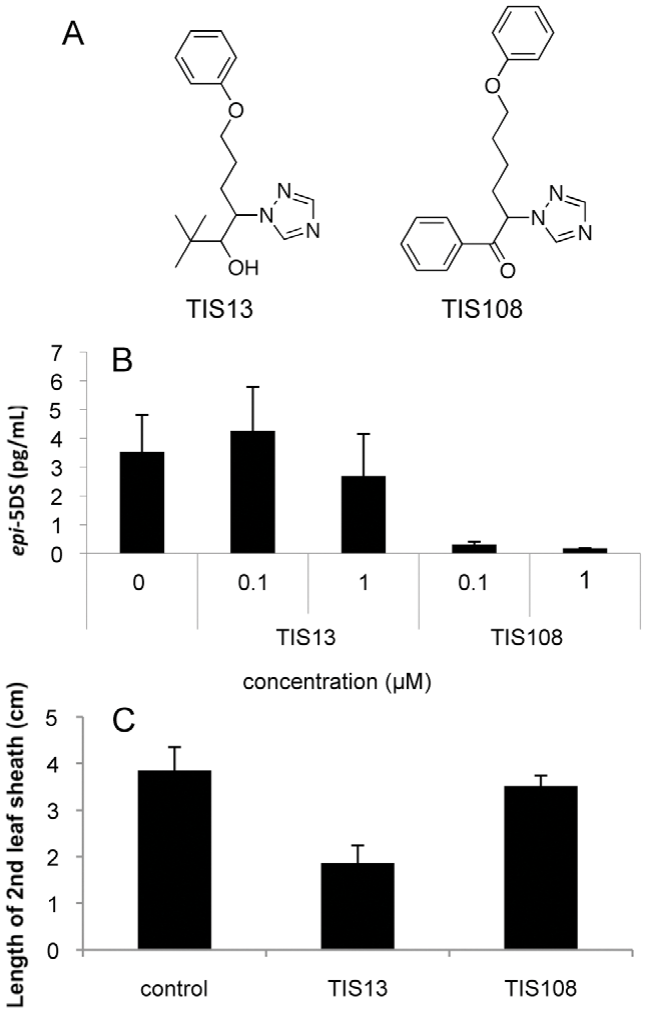
A comparison of the effects caused by TIS13 and TIS108 treatment. (A) Chemical structures of strigolactone biosynthesis inhibitors. (B) *epi*-5DS levels in root exudates of chemical-treated rice seedlings determined by LC-MS/MS. The data are means ± SD of three samples. (C) The length of 2nd leaf sheath in 10 µM chemical-treated rice. The data are means ± SD of six samples.

To determine the ability of these chemicals to inhibit SL biosynthesis and cause dwarfism, we analyzed the level of *epi*-5DS, a major endogenous SL in rice seedlings, in root exudates by LC-MS/MS and measured the length of the second leaf sheath in rice (*Oryza sativa* L. cv. Shiokari). SL analysis was performed according to a previous report [13]. With the exception of TIS103 which possesses a 4-fluorophenyl group, TIS derivatives with a halogenated phenyl group (TIS105-TIS107) reduced *epi*-5DS levels in root exudates more effectively than did TIS13, but they were as active as TIS13 in causing dwarfism (Fig. 2). These chemicals also inhibited root growth, which weakens their potency as specific SL biosynthesis inhibitors (data not shown). Although SLs control root morphology such as root hair elongation and lateral root development in Arabidopsis [15], [16], these differences between wild-type and SL-deficient mutants in rice were not observed under our growth conditions. Therefore, the inhibition of root growth by TIS105–TIS107 could be ascribed to their side effect other than inhibition of SL biosynthesis. TIS102, in which the hydroxyl group of TIS13 is substituted with a keto group, was more active in reducing the level of *epi*-5DS in root exudates than TIS13, and exhibited a weak reduction in shoot growth (Fig. 2). TIS101, in which the *tert*-butyl group of TIS13 is substituted with a phenyl group, and TIS104, which has an extended carbon chain, reduced the level of *epi*-5DS in root exudates more effectively than TIS13, but they were as active as TIS13 in causing dwarfism (Fig. 2). These results suggest that TIS derivatives that have a keto group rather than a hydroxyl group, a phenyl group instead of a *tert*-butyl group and an extended carbon chain might be more potent and specific SL biosynthesis inhibitors. Based on this notion, we synthesized TIS108 (Fig. 3A). TIS108 was 100-fold more active in reducing the level of *epi*-5DS in root exudates and less active in causing dwarfism than TIS13 (Fig. 3B and C). Eventually, TIS108 was the most potent and specific SL biosynthesis inhibitor among the chemicals we synthesized. Our initial preparation of TIS108 was a racemic mixture. To determine the effect of each enantiomer on reducing the level of strigolactone, we separated TIS108 into two enantiomers by using a ChiralCel OJ column (Daicel Chem. Ind., Osaka, Japan. mobile phase; *n*-hexane: 2-propanol = 7∶3, v/v). We found that both enantiomers were equally effective in reducing the level of *epi*-5DS in root exudates in comparison with racemic TIS108 (data not shown).

### Analysis of SL levels in TIS108-treated rice

**Figure 4:**
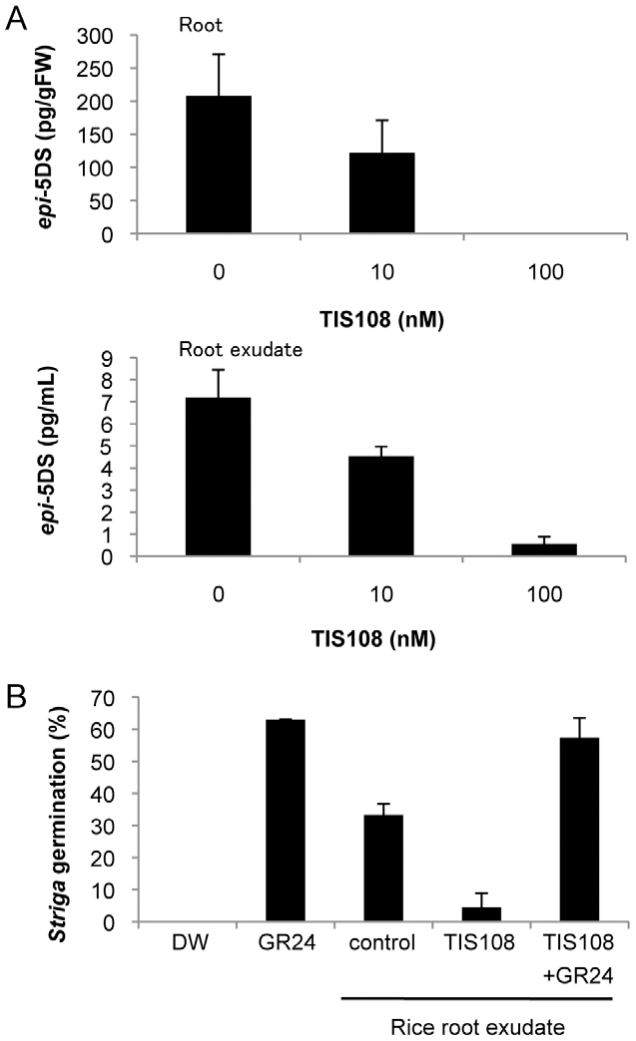
Effects of TIS108 on 2-week-old rice seedlings. (A) *epi*-5DS levels in roots and root exudates of TIS108-treated rice seedlings determined by LC-MS/MS. (B) Germination stimulant levels in culture media of 1 µM TIS108-treated rice seedlings using *Striga* seeds. DW, distilled water; GR24, 1 µM GR24; control, non-treated rice culture media; TIS108, culture media of 1 µM TIS108-treated rice; TIS108+GR24, culture media coincubated with 1 µM GR24 and 1 µM TIS108. The data are means ± SD of three samples.

To exclude the possibility that TIS108 inhibits SL export and reduces the level of *epi*-5DS in root exudates, we analyzed endogenous *epi*-5DS levels in rice roots. The data clearly indicates that TIS108 strongly reduced the levels of *epi*-5DS in both roots and root exudates in a dose-dependent manner at the concentration ranges of 10–100 nM (Fig. 4A). This result suggests that TIS108 inhibits SL biosynthesis in rice.

### Striga germination assay

SLs are not only branching inhibitors but also seed germination stimulants for the root parasitic weeds *Striga* and *Orobanche* [1]. We used a highly sensitive germination assay using *Striga hermonthica* seeds to evaluate TIS108 in the control of root parasitic weed germination [17]. Rice root exudates were collected according to a previous report [13]. In agreement with the results of *epi*-5DS analysis by LC-MS/MS, the culture media of TIS108-treated rice seedlings contained less germination stimulating activity than those of control plants (Fig. 4B). The reduced germination stimulating activity of TIS108-treated root exudates is not due to a direct inhibition of *Striga* germination by TIS108, because an addition of 1 µM GR24 to the root exudates from TIS108-treated plants restored *Striga* germination (Fig. 4B). These results indicate that TIS108 could potentially be applied to reduce germination of root parasitic weed seeds.

### Concluding remarks

To develop more potent and specific SL biosynthesis inhibitors than TIS13, we carried out a structure-activity relationship study and found TIS108 as the most potent and specific SL biosynthesis inhibitor among the chemicals synthesized in our laboratory. Germination stimulation activity of root exudates from TIS108-treated rice was also decreased possibly due to the reduction of SL-levels by the inhibition SL biosynthesis. As root parasitic weeds are responsible for large-scale crop devastation all over the world, TIS108 can be a new tool for controlling parasitic weeds by the application as root parasitic weed control agent. Jamil et al. demonstrated that several carotenoid inhibitors reduce strigolactone production and *Striga hermonthica* infection in rice and can be an efficient technology to control harmful parasitic weeds [18]. Similarly, SL biosynthesis inhibitors can be new chemical tools to control parasitic weeds and it will be necessary to develop an efficient method for the application of the inhibitor, such as irrigation or spray application. Since SLs are also known to be symbiotic signals on AM fungi and shoot branching inhibitor, it is necessary to verify the effects of TIS108 on these functions [19].

There are many P450s involved in plant growth and development such as gibberellin and brassinosteroid (BR) biosynthesis. Therefore there is the possibility that TIS108 may affect some of the P450s involved in their biosynthesis pathways and reduce plant height. Rice is very sensitive to GA biosynthesis inhibitors such as paclobutrazol or uniconazole-P and reduces plant height when treated with these inhibitors at 500 nM [20]. The length of the rice coleoptile is also affected by the endogenous level of BRs. However, TIS108 causes almost no dwarfism even at 10 µM, as shown in Fig. 3C, and did not decrease the length of the rice coleoptile (data not shown). These results clearly indicate that TIS108 does not inhibit both GA and BR biosynthesis at 1 µM, at which concentration TIS108 clearly reduces the level of SL in rice. On the other hand, TIS13 induced dwarfism at 10 µM, perhaps due to the inhibition of GA biosynthesis. In other words, TIS108 is the most specific SL biosynthesis inhibitor among the tested compounds.

TIS108 reduces the level of SL, but its target site(s) is still unknown. Given the fact that at least one P450 (MAX1) is involved in SL biosynthesis in Arabidopsis and that there are five MAX1 homologues in rice, the target site(s) of TIS108 could be MAX1 homologues. However, several steps in the SL biosynthesis pathway still have to be elucidated [7] and there may be other P450s involved in SL biosynthesis. In the future, it will be necessary to identify the target site of TIS108.

In this study, we demonstrated that TIS108 is a potent SL biosynthesis inhibitor. However, as there still remains a lot of room for structural modification of this chemical group, further studies on structure-activity relationships will lead us to find more potent and specific SL biosynthesis inhibitors. In addition, the results of this study will contribute to design new SL inhibitors.

## Materials and Methods

### Chemicals

GR24 was synthesized as previously described [21] to give four stereoisomers. We used (±)-(3aR*,8bS*,2′R*)-GR24 with the same relative stereochemistry as (±)-strigol.

### Synthesis of TIS13 derivatives

TIS13 derivatives were synthesized essentially as reported [14].

6-phenoxy-1-phenyl-2- (1*H*-1,2,4-trizol-1-yl) hexan-1-one (9 : TIS108): 1-phenyl-2-(1*H*-1,2,4-triazol-1-yl)ethanone (1.3 g) and sodium hydride (0.4 g) were dissolved in dimethylformamide (4 mL) and stirred for 30 min, and then (4-chlorobutoxy)benzene(1.85 g)was added under nitrogen atmosphere and stirred for 12 h at room temperature. The reaction mixture was quenched with distilled water. The aqueous layer was extracted three times with ethyl acetate. The organic layer was dried with NaSO_4_ and concentrated under reduced pressure. The resulting oil was purified by column chromatography (*n*-hexane: ethyl acetate = 3∶2, v/v). The product was isolated as a white solid in 12.7%. ^1^H NMR (CDCl_3,_ 500 MHz) δ1.43–1.61 (2H, m), 1.75–1.90 (2H, m), 2.15–2.36 (2H, m), 3.92 (2H, t *J* = 6.3 Hz), 6.09 (1H, dd *J* = 4.8, 10.3 Hz), 6.83 (2H, d *J* = 9.0 Hz), 6.93 (1H, t *J* = 6.8 Hz), 7.26 (2H, t *J* = 8.0 Hz), 7.51 (2H, t *J* = 6.5 Hz), 7.63 (1H, t *J* = 6.5 Hz), 7.95 (1H, s), 7.99(2H, d *J* = 8.3 Hz), 8.38 (1H, s); ^13^C NMR (CDCl_3_, 300 MHz) δ 194.2, 158.8, 151.4, 143.0, 134.4, 134.3, 129.5, 129.2, 128.8, 120.8, 114.4, 67.1, 63.9, 32.7, 28.6, 22.8; HRMS (ES+) calcd. for C_20_H_23_N_3_O_2_^+^ (M+H) 336.1712, found 336.1707; Melting Point 107–108°C.

### Rice hyproponic culture

We used rice normal cultivar, Shiokari. Rice seeds were sterilized in 2.5% sodium hypochlorite solution containing 0.01% Tween 20 for 30 min, rinsed with sterile water and incubated in sterile water at 25°C in the dark for 2 days. Germinated seeds were transferred into hydroponic culture media [4] solidified with 0.6% agar and cultured at 25°C under fluorescence white light with 14 h light/10 h dark photoperiod for 6 days. Each seedlings were transferred to a glass vial containing a 12 ml sterilized hydroponic culture solution and grown under same condition for 6 days. After 6 days, seedlings were transferred to a new glass vial containing a same culture solution with or without chemicals for 1 day.

### SL analysis

SL analysis was performed according to the previously described method [4]. Briefly, the hydroponic culture media (10 ml) were extracted with ethyl acetate twice after adding d1-*epi*-5DS (200 pg) as an internal standard. The organic layer was dried under nitrogen and dissolved in 1 ml ethyl acetate:*n*-hexane (15∶85, v/v). The solutions were loaded onto Sep-Pak Silica 1 ml cartridge (Waters Corp., Massachusetts, USA), washed with same solution twice, eluted with ethyl acetate:*n*-hexane (35∶65, v/v) three times and concentrated *in vacuo*. The roots were homogenized in acetone containing d1-*epi*-5DS (200 pg). The filtrates were dried under nitrogen and dissolved in water. The solutions were extracted with ethyl acetate twice, dried and dissolved in 10% acetone. The extracts were loaded onto Oasis HLB 3 ml cartridges (Waters), washed with water twice, eluted with acetone twice and dried under nitrogen. The concentrates were dissolved in 1 ml ethyl acetate:*n*-hexane (15∶85, v/v) and loaded onto Sep-Pak Silica 1 ml cartridge, washed, eluted and concentrated to dryness. The *epi*-5DS-containing fractions from culture media and roots were dissolved in 50% acetonitrile and subjected to LC/MS-MS analysis.

### Striga germination assay

Germination assay using *S. hermonthica* was performed as described previously [17]. For bioassay, de-ionized water and GR24 solution were used as negative and positive controls, respectively.
